# Parents’ wellbeing: perceptions of happiness and challenges in parenthood in Latin America

**DOI:** 10.1080/17482631.2025.2454518

**Published:** 2025-01-20

**Authors:** Angel Urbina-Garcia

**Affiliations:** School of Education and Social Sciences, University of Hull, Hull, UK

**Keywords:** Parental wellbeing, Latin American parents’, happy parents, parenthood, Parents’ Machismo/Marianismo

## Abstract

**Introduction:**

Traditional research on parenthood and wellbeing often employs a positivist perspective and focuses on non-LA samples -limiting our knowledge and understanding of the influence of cultural components such as Machismo and Marianismo, have in parents’ wellbeing. This study explored how Latin American (LA) parents’ wellbeing is influenced by parenthood in a culture strongly influenced by such gender-based perspectives.

**Methods:**

An interpretative perspective was employed to qualitatively explore fifteen LA parents’ lived experiences and data were analysed via Thematic Analysis. The American Psychological Association’s Journal Article Reporting Standards for Qualitative Research (JARS-Qual), was followed to compile this paper.

**Results:**

Results showed that socio-economic factors such as crime, violence, and economic inequality, negatively influence parents’ wellbeing -emotions experienced and life satisfaction.

**Discussion:**

Parents find joy in sharing own personal experiences with their children and passing on gender-based models. However, parents perceive family pressure as a “burden” when expected to follow principles of Machismo/Marianismo. Similar to Asian, but unlike European parents, LA parents experience a mixture of positive and negative emotions whilst parenting -shaped by Machismo and Marianismo. This study makes a unique contribution by uncovering the unique influence of LA socio-economic challenges and cultural impositions and expectations and its influence on parental wellbeing.

## Introduction

Subjective well-being (SWB) is a cornerstone concept within the field of positive psychology. Pioneering research by Ed Diener and colleagues (Diener et al., Geerling & Diener, 2020; Weiss et al., 2013) define SWB as a multifactorial construct considering an individual’s cognitive and affective evaluations of their life. The cognitive component refers to a person’s judgements about their life satisfaction, both globally and in specific domains such as relationships, work, or health (Cankardas et al., 2024). The affective component focuses on the emotional experiences associated with life, including both positive emotions (e.g., joy, contentment, love) and negative emotions (e.g., sadness, anger, anxiety) (Merkle et al., 2024). These cognitive and affective elements interact dynamically to influence an individual’s overall sense of wellbeing (Diener & Seligman, 2004; Yoo et al., 2018). Parenthood, on the other hand, is another multifactorial construct comprising a range of roles, responsibilities and experiences associated with raising and caring for children (Cabrera et al., 2018). Recent psychological research has highlighted the dynamic nature of parenthood, emphasizing its developmental significance and diverse socio-cultural contexts. Furthermore, parenthood entails significant psychological adjustments and adaptations as individuals navigate the challenges and rewards associated with raising children (Nelson et al., 2014). This includes managing stressors, coping with uncertainties and experiencing personal growth and development through the parenting journey (Nelson-Coffey & Stewart, 2019; Prime et al., 2020). Parenthood seems to be influenced by a range of factors, however recent psychological research has also highlighted the major role that parenthood plays in shaping parents’ wellbeing.

The link between parenthood and subjective wellbeing (SWB) has been investigated extensively with an increasing body of international research showing that parenthood is as a multifactorial experience, capable of causing both positive and negative consequences for parents (Rado, 2020; Waterman, 2017). Longitudinal studies have provided a wealth of empirical evidence suggesting a positive association between parenthood and SWB (Aassve et al., 2015; Balbo & Arpino, 2016; Frijters et al., 2020; Pollmann-Schult, 2014). However, the cross-sectional studies seem to show a contrasting picture, revealing negative correlations (Hansen & Slagsvold, 2012; McLanahan & Adams, 1987; Myrskylä & Margolis, 2014; Stanca, 2012). A potential explanation for these seemingly contradictory findings lies in the limited geographical scope of the research. The majority of the studies were conducted in European countries (Sheldon & Lucas, 2014), potentially limiting their generalizability to populations in Latin America or Asia. Furthermore, inconsistencies in the reported effects may be attributable to the potential mediating role of biopsychosocial factors (Myrskylä & Margolis, 2014; Senior, 2014). These factors, which can significantly influence the parent–child relationship, have often been overlooked in previous research (Kohler, 2015; Myrskylä & Margolis, 2014; Rado, 2020).

The influence of parenthood on subjective wellbeing (SWB) is undoubtedly complex and potentially mediated by a multitude of factors (Clark et al., 2008; Kohler et al., 2005). For example some factors comprise the number of children a parent has, parents’ personality traits, and the broader context surrounding their lives (Aassve et al., 2015; Clark et al., 2008; Kohler et al., 2005). Myrskylä and Margolis (2014) highlight the significant variations in parental SWB based on socioeconomic status, gender, marital status, and environmental context. Personality and gender are posited as relevant factors that influence parental SWB (Myrskylä & Margolis, 2014). Socioeconomic status and culture are also suggested to play a mediating role (Aassve et al., 2015). Interestingly, longitudinal studies further reveal a decline in SWB as children age (Balbo & Arpino, 2016). Research suggests an eventual return to pre-parenthood SWB levels once children reach adolescence (Frijters et al., 2011). However, this trend is not universally observed. Other studies report a sustained positive impact of parenthood on SWB in the long term (Mikucka, 2016; Pollmann-Schult, 2014). While existing research, particularly from Europe, has provided valuable evidence around these mediating factors, a geographical limitation exists. The existing body of European research certainly aids our understanding of this relationship; nevertheless, we have a limited knowledge and understanding of parenthood and SWB in the Latin American culture which is heavily influenced by cultural expectations and roles deeply rooted in Machismo and Marianismo.

### Subjective wellbeing in Latin America: the socio-cultural context

Latin America (LA) represents a vast and populous region, encompassing nearly 600 million inhabitants (Rojas, 2016). It is also recognized as the world’s most unequal continent economically (Rojas, 2016). Beyond economic disparity, however, LA is characterized by a shared cultural heritage, including distinctive worldviews, cultural practices, and values (Inglehart & Carballo, 1997; Larrain, 2013; Veliz, 2014). While each nation within LA has developed its own unique cultural identity (Veliz, 2014), cross-cultural research suggests that a core “*Latin American culture*” transcends individual countries (Larrain, 2013). This shared culture is believed to be rooted in common social practices and values embedded within the broader geographic region, rather than solely in national identities. Evidence supporting this notion comes from studies such as the World Values Survey (World Values Survey, 2020), which demonstrates comparable value systems across nations like Chile, Argentina, Mexico, and Brazil. As a result, it is crucial to acknowledge both the diversity and the unifying elements within the Latin American context to better understand how parenthood is shaped by these cross-cultural similarities and differences on a broader scale.

Research consistently highlights the prevalence of a “Macho culture” or “Machismo” in Latin American (LA) countries (Flake & Forste, 2006; Girman, 2013). This system of beliefs emphasizes masculine strength, aggression, self-sufficiency, hypersexuality, and other gendered practices that denote male dominance over women across various aspects of life, including job opportunities, pay gaps, parenting roles, and access to social activities (e.g., Girman, 2013). Within this framework, parenthood seems to be significantly impacted by gender expectations. Women are traditionally viewed as the primary caregivers, responsible for child-rearing without questioning the man’s role (Girman, 2013, Hardin 2002; Koenig & Eagly, 2014). The male role, conversely, is often associated with being the “breadwinner” (Girman, 2013; Hardin 2002). Further, shaping parental practices are the expectations associated with “Marianismo,” the ideal of Latino females being submissive, humble, and self-sacrificing with a focus on nurturing the family (Castillo et al., 2010; Kosmicki, 2017). Recent research suggests that machismo and marianismo significantly influence parental practices by reinforcing existing gender roles and a “patriarchal” way of life (Brumley, 2013; Rojas, 2016; Taylor et al., 2019). However, to our knowledge, there is no research exploring the influence of such gender-based roles in parents’ wellbeing in LA samples. This is crucial given the increasing body of research showing a substantial shift in modern societies whereby family structures and dynamics are changing in the twenty-first century (Bower-Brown, 2022) specifically around males, females and now, non-binary roles (Worthen & Herbolsheimer, 2022). Noteworthy, cultural factors such as Marianismo and Machismo have been studied in different contexts in previous research, for example, Nuñez et al. (2016)investigated the association between Marianismo/Machismo and Cognitive-Emotional factors across cities in the US including, New York, Chicago, Miami and California. Findings revealed that a higher adherence to the principles of Marianismo/Machismo was significantly associated with higher level of negative cognitions and emotions. A recent systematic review of the literature found that women scoring high in Marianismo traits in Latin American and non-Latin American countries, engaged in practices that deteriorated their mental health – for example, they tended to practice unsafe sex, accept partner violence and experience higher levels of depression, thus negatively impacting their overall wellbeing (Palmer et al., 2024). Sanchez et al. (2018) found that Latino women identifying themselves with the Marianismo-like attitudes and traits, experienced more discrimination, which led to experience higher levels of depression. Taken together, these cross-cultural studies highlight the pervasive impact of machismo and marianismo on psychological well-being across diverse populations, emphasizing the need for additional research that could lead to culturally sensitive mental health interventions that address this gender-driven impact.

LA parents are faced with a range of socioeconomic challenges that significantly impact their lives and parenting experiences. Some challenges include high levels of crime and violence (Kosmicki, 2017) and drug-related problems (Carpenter, 2014). These social issues exert a powerful influence on parents at the individual level, shaping their parenting practices (Liu et al., 2017) and ultimately impacting their happiness and overall subjective wellbeing (SWB) (Rojas, 2016). Existing research on the relationship between socioeconomic status (SES) and parental SWB in LA shows a complex picture. While some studies reveal that parents from lower SES backgrounds report lower levels of happiness and SWB (Chindarkar, 2014), other studies suggest a contrasting trend, with parents reporting higher levels of happiness and SWB compared to their more affluent counterparts (Rojas & García-Vega, 2017). Crime and violence, prevalent psychosocial problems in many LA countries, have been associated with lower parental SWB (Alfaro-Beracoechea et al., 2018). Conversely, economic stability is often linked to higher reported levels of SWB (Schnettler et al., 2021). However, the research also indicates that even parents living in poverty report unexpected findings of happiness and SWB (Rojas & García-Vega, 2017; Terrazas-Carrillo et al., 2016). Gender differences have also been observed in empirical research. For example, a study by Terrazas-Carrillo et al. (2016) suggests that job satisfaction, being male, and having only one child are positively associated with parental happiness and SWB in LA. Conversely, the burden of overwhelming household tasks disproportionately falls on women, negatively impacting their happiness and wellbeing. Nevertheless, studies are needed to extend our knowledge and understanding of how modern family dynamics and structures in the 21^st^ century, are not only changing but also impacting parental roles, practices, behaviours and more importantly, parents wellbeing.

### International research on the perceptions around parenthood: Is Latin America included?

A critical review of the literature reveals a significant gap in our understanding of the relationship between parenthood and SWB in LA culture heavily shaped by Machismo and Marianismo (Conceição & Bandura, 2008; Rojas, 2016). Recent systematic reviews show a trend of research primarily focused on cultures in developed regions such as like North America, Australasia, and Europe, with scant attention paid to Latin America or Africa. Additionally, these studies reveal an overreliance on standardized measures and a disregard for cultural context (Concienciao & Bandura, 2008; Rojas, 2016). This geographical bias and methodological trend, have limited our knowledge in two essential ways. Firstly, despite the wealth of research from the aforementioned developed regions, scant attention has been devoted to exploring the lived and personal experiences of parents around parenthood and their wellbeing. The prevailing positivist perspective, which prioritizes objective measurement, has inadvertently marginalized the narratives and subjective realities of parenthood in Latin American parents. Consequently, our understanding of the interplay between parenthood emotions, wellbeing and a Machismo/Marianismo-led culture, remains incomplete. Secondly, whether approached from a positivist or interpretivist standpoint, research into parenthood within Latin American samples are scarce. A brief analysis of international research supports this point.

Tao’s (2005) analysis of data from the Taiwan Panel Study of Family Dynamics, for instance, found no association between the number of children and parental happiness and wellbeing. However, this study solely relied on standardized scales, failing to capture participants’ lived experiences and personal interpretations around Taiwanese cultural influence. Similarly, Kohler et al. (2005) investigated a large dataset of monozygotic twins, concluding that the first child significantly affects parental happiness, while subsequent children do not. However, their use of predetermined variables within a large dataset overlooks the potential influence of cultural practices, social values, and religious beliefs on the subjective experience of parenthood. Other studies by Frey and Stutzer (2006) and Haller and Hadler (2006) employed standardized measures and found a strong correlation between parenthood and wellbeing. However, these studies failed to explore how social pressures, such as those exerted by family members, may influence parenting practices for both men and women and how these may influence their wellbeing. Hansen et al. (2009) analysed individual-level data from Norway, revealing no association between motherhood and SWB. Interestingly, childless women reported lower self-esteem and life satisfaction. Limitations of this study include failing to acknowledge sociocultural factors shaping parenthood, such as gender Norwegian roles and family income, from the participants’ perspectives. A concerning finding from our review is the apparent absence of qualitative studies conducted in LA that explore parental perceptions. While literature reviews by Rojas (2016) claim to focus on happiness and wellbeing in LA, their focus limited to areas such as romantic relationships, social inequality, poverty and GDP. Only one relevant study by Velasquez (2016) was identified. This qualitative Colombian study suggests that access to various forms of parenting support enhances SWB for parents, with participants reporting that their children’s health and education are the most significant factors influencing parents’ satisfaction. However, only Colombia was considered which limits our understanding of how family dynamics and culture principles around Marianismo and Machismo in other LA countries, influence parent’s wellbeing.

Taken together, the scientific literature seems to have limited evidence around parents’ experiences of the way in which parenthood is influenced by specific socio-cultural factors such as Machismo and Marianismo. Furthermore, limited research with LA samples clearly suggests the need for additional studies in LA overall. Moving beyond standardized measures and incorporating qualitative methodologies seems to be crucial for achieving a more comprehensive understanding of parenthood and SWB in this understudied region.

### The state of the art of subjective wellbeing

The seminal work of Ed Diener (1984) led to a surge of research interest in subjective well-being (SWB), shaping our contemporary understanding of the construct. Within the scientific community, a consensus has emerged that SWB encompasses three core elements: life satisfaction, frequent positive emotions, and infrequent negative emotions (Diener, 1984; Diener & Seligman, 2004; Diener et al., 1999). Consequently, much of the research on parenthood and wellbeing has focused on measuring these specific components (Milfont et al., 2020). However, more recent research suggests limitations in prior studies that solely relied on measuring happiness (Diener’s model) through an individual’s cognitive self-assessment via self-report (Mureșan et al., 2023; Velasquez, 2016). This approach is susceptible to personal biases. Cross-cultural researchers call for the need to examine all three elements of SWB to achieve a more comprehensive understanding (Yin et al., 2023).

A eudaimonic perspective, however, seems to challenge the adequacy of Diener’s model in capturing the full complexity of wellbeing. Stemming from Aristotle’s notion of “eudaimonia” (the highest human good), suggests that true wellbeing may not solely reside in happiness, self-satisfaction, or positive emotions (Ryff, 2013). This challenges Diener’s emphasis on life satisfaction and positive emotions. The eudaimonic approach underscores the importance of examining individuals’ striving aspects, their meaning-making in life, and self-realization, aspects which are heavily shaped by cultural norms and values (Huta & Waterman, 2014; Ryff, 2013). Research suggests that data on ageing, personality, family experiences, and other life engagements provide evidence that eudaimonic wellbeing may significantly impact overall health and wellbeing (Ryff, 2013). These factors are not fully considered in Diener’s model and warrant further investigation, as wellbeing is fundamentally linked to how individuals perceive and navigate life’s challenges. Accordingly, exploring how parents create meaning in their experiences shaped by gender-based cultural expectations, particularly amid the challenges of parenthood, becomes essential.

Life satisfaction, as a core component of SWB, is widely understood as a cognitive self-assessment of one’s life (Mulet, 2020). However, the realm of affect encompassing emotions, presents a more complex challenge. Researchers have proposed various categories to capture the complexity of emotions (Izard, 2013). Watson and Tellegen’s influential work sought to illuminate the structure of affect, consistently identifying two core bipolar dimensions: positive and negative affect (Smillie et al., 2015). Their Circumflex Theory posits that positive and negative emotions are distinct and orthogonal factors (Rubin & Siegler, 2004; Watson & Tellegen, 1985; Watson et al., 1988). Positive affect, according to this theory, is characterized by positive appraisal, while negative affect is associated with distress or feelings of upset. Despite its contributions, Watson and Tellegen’s model has been critiqued for primarily relying on studies conducted with WEIRD (Western, Educated, Industrialized, Rich, and Democratic) populations (Barrett, 2017). This WEIRD-centric approach limits our understanding of affect across diverse socio-cultural contexts. Furthermore, a eudaimonic perspective emphasizes that emotions are self-constructed experiences rooted in individual understandings and interpretations (Greco & Stenner, 2013). Since culture shapes social constructs, the very definition of emotions can vary and seem to be culturally dependant. Consequently, the physiological and psychological experience of a given emotion, such as those experienced by parents, may differ across cultures (Greco & Stenner, 2013; Urbina-Garcia, 2024). Additionally, while Watson and Tellegen acknowledged the role of genetics in affect, critics argue for a more in-depth exploration of the precise neurophysiological mechanisms underlying emotional experience (Cloninger et al., 2015). More importantly, their initial model gave limited consideration to socio-cultural aspects, which were only partially addressed by subsequent research. These limitations suggest the need for additional research on parenthood and SWB by moving beyond the confines of the WEIRD lens and integrate an eudaimonic perspective that acknowledges the cultural construction of emotions in non-WEIRD nations.

An alternative perspective on wellbeing posits its division into two key categories: hedonia and eudaimonia (Thorsteinsen & Vittersø, 2018). This framework suggests that capturing well-being requires measuring concepts from both domains. A recent systematic review by Huta and Waterman, (2014) supports this notion by differentiating hedonic and eudaimonic well-being. Hedonic wellbeing encompasses feelings of satisfaction, enjoyment, and pleasure, while eudaimonic wellbeing focuses on personal growth and self-realization. However, this approach is not without limitations. First, the experience of pleasure is demonstrably influenced by cultural norms, social practices, and societal values (Kringelbach & Berridge, 2017). Consequently, what constitutes pleasurable experiences for parents may vary considerably across cultures. Second, eudaimonic wellbeing is also susceptible to the influence of contextual factors. The meaning individuals ascribe to self-actualization can differ significantly (i.e., influenced by socio-cultural expectations), further complicating its measurement and exploration (Fowers, 2016). Overall, the above studies suggest that SWB is influenced by parents’ self-evaluation which seems to be shaped by cultural factors.

### Parenting in Latin American and its impact on subjective wellbeing: a rationale

A critical review of the literature on parenthood and SWB reveals a significant geographical bias and a limited focus on exploring strong LA-based gender-based roles such as Machismo and Marianismo around parents’ wellbeing. The review of the scientific literature revealed that research has focused on parents in the United States, Europe, and Australasia, with a scarcity of studies investigating LA populations (Concienciao & Bandura, 2008; Rojas, 2016). Furthermore, the existing research has primarily relied on quantitative methodologies to measure constructs derived from Diener’s model (Diener, 1984). While valuable, this approach leaves ample room for future research that incorporates a meaning-making perspective. Our review highlights a critical gap in our understanding of how Latin American parents define and conceptualize parenthood in light of cultural values and practices influenced by Machismo and Marianismo. This knowledge is essential for elucidating how these conceptualizations, in turn, influence parental wellbeing, not only in the context of current socioeconomic conditions in LA but also their strong culture around Machismo and Marianismo. Simultaneously, LA countries generally face a number of challenges compared to most industrialized nations (Worldbank, 2020). These include lower GDP per capita, lower levels of educational attainment and social mobility, and higher rates of crime, violence, and drug use (UNICEF, 2022; Worldbank, 2020). Exploring how these socio-cultural conditions influence parents’ notions of parenthood and how these notions, in turn, affect their wellbeing seems to be needed to expand our understanding of the relationship between parenthood and SWB. Additionally, the Circumflex Theory by Watson and Tellegen suggests that positive and negative emotions can indeed co-exist simultaneously – which is particularly relevant when considering the adverse circumstances parents live with, in Latin America. Diener’s model of Subjective Wellbeing, on the other hand, suggests that wellbeing is not only about experiencing emotions, but also about factors that contribute to life satisfaction such as parenthood. We were interested in understanding how parenthood could be a factor that contributes to life satisfaction as well. Therefore, this study aimed to address these gaps by exploring the perceptions of Latin American parents as to how their wellbeing is influenced by parenthood shaped by Marianismo and Machismo. Our study was guided by four research questions as follows: a) What are perceptions of parents around becoming a parent? b) What do parents perceive as the challenges during parenthood in Latin America? c) What do parents perceive as pleasurable experiences during parenthood and what children’s behaviours make them feel good about being a parent? d) What are the perceptions of parents as to how challenges/pleasurable experiences influence their wellbeing? As a result of the review of the psychological literature and the aim of our study, our primary hypothesis reads as follows: *Parenthood among Latin American individuals in Latin America is experienced as a complex interaction of positive and negative emotions, significantly shaped by the socio-economic challenges and cultural pressures related to Machismo and Marianismo*.

## Methodology

### Design

We followed a qualitative approach to explore the lived experiences of parenthood and its influence in parents SWB (Coolican, 2017; Hammarberg et al., 2016). This helped us to prioritize an in-depth understanding of the richness and complexity of participants’ personal experiences. We also used a cross-sectional approach, collecting data at a single point in time. We recognize the inherent subjectivity of qualitative research, acknowledging the potential for researcher bias to influence data interpretation. To mitigate these limitations, methodological rigour was maintained throughout the research process, including member checking and triangulation techniques. Nevertheless, the team agreed that the advantages of the research design outweighed its limitations and which helped to address the main aim of the study.

### Participants

To ensure the research addressed the central research question, a purposive sampling strategy was employed (Crabtree & Miller, 2022). Additionally, the literature suggests that qualitative samples ranging from 6 to 22 participants are adequate when data saturation is achieved (Hammarberg et al., 2016). Hence, our purposive sampling was used to recruit a sample of 15 participants, maximizing variation in viewpoints and experiences. We used the following inclusion criteria:

- Parents born and currently residing in LA countries to ensure cultural influences on the experience of parenthood were captured.
- Parents with at least one child between the ages of 4 and 10 years old – allowing exploration of parental wellbeing informed by at least 4 years of parenting experience.
- Parents of children without reported disabilities or mental disorders -to ensure our primary focus remained on the influence of “typical” parenthood on wellbeing.

Participants were excluded if they:

- Did not have children between the ages of 4 and 10.
- Resided in a non-Latin American country.
- Parents not currently living in Latin America.
- Self-reported mental health problems or ongoing psychological treatment.

The administrators of two Facebook groups were contacted – groups which included approximately 1000 members altogether – where Latin American parents tend to seek help and/or information related to effective parental practices. Once permission was granted by administrators, the study Ad was then posted in both Facebook groups. Participants interested contacted the researchers via email and thus, we shared Consent Forms and Participant Information Sheets. Once the consent form was signed by participants a mutually convenient time was agreed to hold the interviews via TEAMS or Zoom. Prior to and during the interview, participants were reminded that they could stop the interview without giving any explanation or withdraw their data without any explanation. Once the interviews finalized, we thanked participants and shared a debriefing document with useful links to mental health organizations.

Participant demographics are presented in Table 1.

**Table 1.** Parents demographics.

Participants	No. of children	Age of children	Gender of children	Country of Origin	Country of Residence
Parent F1	1	4	Female	Chile	Chile
Parent F2	2	4 & 7	Male/Female	Mexico	Mexico
Parent F3	1	6	Male	Colombia	Colombia
Parent F4	1	5	Male	Ecuador	Ecuador
Parent F5	1	6	Female	Mexico	Mexico
Parent F6	1	4	Female	Costa Rica	Costa Rica
Parent F7	2	5 & 8	Male	Ecuador	Ecuador
Parent F8	3	3, 6 & 9	1Male/2Female	Honduras	Honduras
Parent M9	1	5	Female	Colombia	Colombia
Parent M10	1	10	Male	Colombia	Colombia
Parent M11	3	1, 3 & 5	1 Male/2 Female	Peru	Peru
Parent M12	2	1 & 9	Male	Colombia	Colombia
Parent M13	1	7	Female	Mexico	Mexico
Parent M14	1	8	Male	Argentina	Argentina
Parent M15	1	5	Female	Costa Rica	Costa Rica

*Note*: F and M are used to denote self-identified male/female parents considered an important clarification given cultural expectations in Latin America.

### Measures

To explore the participants’ personal experiences of parenthood, we employed semi-structured interviews (Buys et al., 2022). This method ensured a balance between flexibility and structure, allowing the interviewer to explore core areas of inquiry while also adapting to the unique narratives of parents (Galleta, 2013). Following a comprehensive review of cross-cultural qualitative research, an eight-question interview schedule was developed. Our interview was also informed by two essential components. First, we drew upon the systematic review conducted by Izzo et al. (2022), which synthesized existing research on children’s happiness. Second, the qualitative protocol of the Friends and Family Interview (FFI; Psouni et al., 2020; Steele et al., 2005) served as a foundational structure. The FFI is designed to explore an individual’s social network and their perceptions of these relationships. By adapting this framework, we aimed to capture parents’ perspectives on their relationships with their children and the shared experiences that contribute to their SWB. An example of a question is as follows: “*What is your experience of activities as a parent that you enjoy most?*” To ensure clarity and effectiveness, the interview schedule was piloted with two participants not included in the final sample. The pilot data further informed refinements to the wording of the questions.

### Data analysis

Thematic Analysis (TA) served as the chosen method for data analysis, specifically the six-stage approach outlined by Braun and Clarke (2022). This method facilitated the identification and development of themes based on parents’ perspectives, aligned with the study’s objectives (Kiger & Varpio, 2020). The six stages were rigorously followed to ensure a systematic and transparent analytical process. We adopted an “artfully interpretative” approach to TA aligned with the interpretivist perspective of the study (see Finlay, 2021). This approach emphasizes reflexivity, critical interpretation, relativism, and a deductive stance. It allowed us to explore how parents’ understandings of concepts and meanings are contingent upon the specific contexts that shape them. In the context of this cross-cultural research, interviews were translated from Spanish to English and then back-translated. This approach, as advocated by Epstein et al. (2015), strengthens the accuracy of transcriptions and translations.

When new data no longer yielded significant new insights or revealed novel perspectives, we considered data saturation to be achieved. This was achieved through a constant comparison, where data from each new participant was examined against previously analysed data. The iterative process involved revisiting transcripts, refining codes, and discussing findings among the research team to validate emerging themes. Member checking was fundamental in this regard wherein participants reviewed summaries of their responses to confirm accuracy and resonance with their experiences. Peer debriefing further enhanced the validity of findings, as research team members met continuously with two international researchers and specialists in the psychology of wellbeing and parenthood – external to the research team – to critically discuss the coding framework and interpretations to ensure rigour and reduce researchers’ bias.

### Procedure

Following ethical approval for this study, we targeted parents residing in Latin American countries via social media. We contacted the administrators of two Facebook groups known for offering support and information on effective parenting practices, with a combined membership of approximately 1,000 parents. Following gatekeeper’s approval, a study advertisement was posted within these Facebook groups.

Interested parents contacted the researcher via email, who then provided them – via email - with the informed consent form and participant information sheet for review and signature. Interviews were scheduled via TEAMS or Zoom at mutually convenient times. Both before and during the interviews, participants were informed of their right to withdraw from the study without offering any explanation. Additionally, their consent was obtained for audio recording (without video) of the interview sessions. To enhance the trustworthiness of the research and mitigate potential researcher bias, interviewers employed a bracketing technique throughout the interview process (Creswell, 2013). This involved taking notes during interviews that captured any interviewers’ judgements or emotional responses triggered by participants’ narratives. By analyzing and categorizing these notes, we were able to identify and set aside potential biases during both the interview and data-analysis stages.

### Researcher’s characteristics and reflexivity

The researcher is a 45-year-old Mexican male academic with extensive international experience in academia, having lived and worked in Europe and Asia for 13 years. The researcher is an expert in children’s socio-emotional development and wellbeing as well as in challenges during educational transitions having worked with children and parents extensively in Mexico. The researcher has taught international students from over 50 countries across Asia, Latin America and Europe. His upbringing in Mexico and immersion in the Latin American culture, including firsthand experience with Machismo and Marianismo that significantly shape gender roles and expectations, provided him with a deep understanding of the cultural dynamics central to this study. However, his extended residence in Europe and adoption of European cultural norms, values and practices, offer a holistic and cross-cultural global perspective on parenthood and wellbeing which shaped his approach to the present study with a critical and reflexive lens.

### Ethics

Ethical approval for this study was granted by the Institutional Review Board of the first author’s affiliated university. Our study rigorously observed the principles of respect, competence, responsibility and integrity of the BPS Ethics Code of Practice (British Psychological Society, 2018) and adhered to the recently updated ethical guidelines of the British Educational Research Association (2024). We allocated codes to each participants, and we use pseudonyms in the data-analysis stage.

We followed the American Psychological Association’s Journal Article Reporting Standards for Qualitative Research (JARS-Qual), as proposed by Levitt et al. (2018) which is used to assess and report qualitative studies with a view to strengthening the research quality of the present study.

## Results

**Figure 1.**
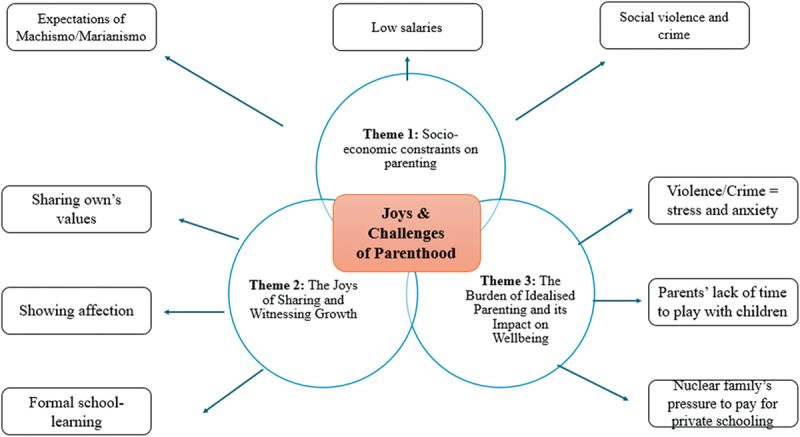
Visual representation of subthemes and main themes identified in the data.

The audio recordings were transcribed verbatim to facilitate thematic analysis. The translated transcripts were then subjected to a rigorous coding process. This process involved systematically assigning codes to segments of data that captured key concepts and ideas (Braun & Clarke, 2022). These initial codes were then grouped into sub-themes, which in turn informed the development of three overarching themes. Table 2 presents an overview of the sub-themes identified during coding, which ultimately led to the formulation of three core themes, whilst Figure 1 shows a visual representation of the themes identified.

**Table 2.** Core themes and sub-themes derived from the Thematic Analysis.

Core Themes	Sub-themes
Theme 1: Socio-economic Pressures and Constraints on Parenting	Subtheme 1: Family/Social pressure and expectations linked to Machismo/Marianismo Subtheme 2: Limiting opportunities for their child’s private schooling and leisure (low paid job) Subtheme 3: Current climate of violence and crime difficulties in future (children getting a god job)
Theme 2: The Joys of Sharing and Witnessing Growth	Subtheme 1: Sharing own’s values with children Subtheme 2: Showing affection (love) to children Subtheme 3: Children growing and learning at school
Theme 3: The Burden of Idealised Parenting and its Impact on Wellbeing	Subtheme 1: Violence and Crime trigger anxiety and stress Subtheme 2: Lack of time of parents’ to provide fun activities. Subtheme 3: Social/Family and Personal pressure to ensure children’s good education (private schooling)

### Theme 1: socio-economic pressures and constraints on parenting

This first theme captures the challenges faced by parents due to the social and economic realities of their LA countries. Participants expressed anxieties related to societal pressures from extended family and friends to provide for their children’s futures, particularly regarding access to high-quality education. There seemed to be a strong pressure on fathers to exercise a Macho attitude (e.g., being more masculine, showing self-sufficiency, bringing more money to home) to provide for their families. Another prevailing sentiment was that private schooling offered a superior education compared to public options. Father’s expected role was to secure a place in a “good” school for their children – as this was seen as essential for ensuring a university degree and a successful career path for their children. Mothers were expected to be in charge of household-related management and get children “ready” for school.

This theme also encompasses the economic hardships experienced by participants. Low salaries and limited job opportunities were reported to restrict opportunities for engaging in leisure activities with their children. Furthermore, concerns about violence and crime in their communities further limited their ability to venture outside the home with their children. These factors combined emerged as significant constraints on parental wellbeing.

Parents reported the expectations from their family whilst parenting:

Yes … there is definitely pressure from my extended family to continue the family lineage … mmm … following in the footsteps of previous generations. They keep reminding me that I am the man of the house and I should provide more money. Their expectation is to have children to preserve the family heritage and name. However, financial obstacles due to low salaries make this a significant challenge. My ability to provide adequately for my children in these economic circumstances concerns me … (Participant M12)

Low-pay jobs seem to make parenting more challenging:

It feels like I’m constantly walking a tightrope. The money I bring home barely covers the basics, let alone sending the kids to private school like everyone says is best. My husband has asked me to stop working and focus on our child. The fees are just astronomical, and there’s no way we can swing them. We just want to give our children the best possible start, but it often comes at the expense of our own happiness. A night out at the movies with friends feels like a luxury we simply can’t afford anymore. It’s tough seeing them enjoy things I can’t offer my family right now. (Participant F1)

Parents pointed out that the social environment around their home, makes their job as parent quite challenging:

These days, letting my child roam the neighborhood with friends feels like taking a huge risk … mmm … I don’t know … the constant worry of muggings or worse, kidnappings, hangs heavy in the air. Every walk down the street feels tense, my eyes scanning for any potential threats. Being a woman and having a young girl, makes me scared of men when going out … .It’s a shame they can’t experience that carefree freedom kids used to have. (Participant F6)

### Theme 2: the joys of sharing and witnessing growth

The second theme highlights the aspects of parenthood that bring participants the greatest satisfaction. A core aspect of this theme is the act of transmitting personal values instilled in participants during their own childhood. Participants described a sense of connection with their own upbringing as they made conscious choices about the values they wished to pass onto their children. The evidence suggests that an intergenerational link appeared to inform their parenting practices.

Physical expressions of affection towards their children were another source of joy for participants. These expressions fostered feelings of happiness and emotional connection. Furthermore, participants expressed gratification in witnessing their children’s intellectual growth, particularly the acquisition of new knowledge from school settings. Similarly, observing their children’s developing self-care skills provided participants with a sense of accomplishment and fulfilment in their parental roles.

Parents enjoy using their experience as children to educate their children:

… mmm … having a child presents a unique opportunity to share the knowledge and values passed down from your own parents […] it also allows for a different parenting approach, potentially moving away from methods you didn’t appreciate in your own upbringing […] such as corporal punishment. This tells me that there is a generational shift in parenting styles … I guess … […] I still need to ensure my three little boys become strong men … […] I feel proud when my three men tell me they will look after their mum as they should … (Participant M11)

Participants use parenting to create an emotional bond with their children:

Kissing my son brings me great joy … in his face, I see a reflection of his mother, and this act of affection allows me to express my love and care for him. However, I am a man, and I cannot show him too much affection cause he can see me as weak. Perhaps it fulfils a need for love I didn’t experience in my own childhood, allowing me to shower my son with the affection I may have missed … (Participant M14)

Parents report that they feel good when witnessing their child being more independent:

… you know … being a mum is difficult, but […] witnessing my child’s development and learning at school brings me a sense of relief. While work commitments limit my time together at home, I take advantage of any opportunity to connect. These moments when I can watch her learning basic skills, like washing her hands or getting dressed independently, provide me with a sense of accomplishment and satisfaction in my role as a parent […] I teach my eldest daughter how to do household duties and “serve” her siblings as this will help her when she gets married … (Participant F8)

Parents report that they feel proud and happy when seeing their children displaying expected behaviours associated with gender-based roles:

… she doesn’t reply to her dad in a bad way … […] she obeys him. I feel happy and relieved when my little girl is kind, nice to others and obedient … she helps her cousins do their homework … and I tell her, good girl! (Participant F5)

When I see my boy playing with his peers or playing football at school or wrestling with his cousins, I can see he is tough and I feel happy that I did not raise a weak boy … […] These moments make me feel proud of my eldest … (Participant M10)

### Theme 3: the burden of idealised parenting and its impact on wellbeing

This included parents’ experiences of how societal pressures to embody the ideal of a “good parent”, negatively affect participants’ subjective wellbeing. Participants described feeling emotionally drained and inadequate in response to constant pressure from extended family members. This pressure often focussed on the expectation that fathers (and sometimes mothers) work tirelessly to earn more money, solely to secure their child’s place in a prestigious public university. This was perceived as the pathway to a successful career for their children and as a successful “man of the house”. These pressures, coupled with the high crime rates in their communities, seem to contribute significantly to participants’ stress and anxiety. The fear of violence and potential harm to their children restricted their ability to engage in outdoor activities as a family, further limiting their enjoyment of parenthood. Emotional strain was also evident in participants’ expressions of sadness regarding the time constraints imposed by work. They lamented the lack of quality time dedicated to their children due to work-related demands. This sense of inadequacy and the inability to fulfil their perceived parental obligations emerged as a significant threat to their overall wellbeing.

Parents report to feel anxious about some situations on their neighbourhood which impacts their wellbeing:

The constant fear of mugging or robbery in certain areas creates significant stress when venturing out with my children, whether to school or for social visits. This is worst since I have two girls and boys are aggressive in the neighbourhood. […] To avoid these dangers, I must carefully navigate and potentially restrict ourselves to safer neighbourhoods. This situation can lead me to having feelings of anxiety and a reluctance to leave the house altogether. However, there is a sense of uncertainty about whether these precautions are truly necessary or an overreaction … we need the man of the house to protect us all … (Participant F2)

Limited time with their children, impacts the wellbeing of participants:

… balancing work and family life can be challenging. The need to hold two jobs to meet household expenses unfortunately limits the time available to spend with my child. This situation can lead to feelings of sadness, particularly when considering missed opportunities for shared activities like homework or simply watching television together. Despite the sacrifices made, there is a sense of uncertainty about whether these efforts are sufficient to fulfil my role as a breadwinner for the family … sometimes I think whether I am enough of a man for my wife and daughter … (Participant M15)

Ensuring a high level of education – to secure a good job – seems to be the main expectation from family members which seems to impact parents’ wellbeing:

… you know, the pressure from my family to enrol my children in a private school is causing me significant stress. Their emphasis on me, securing their future through a prestigious education and a good job feels overwhelming to me […] despite my efforts, I don’t always feel like they are appreciated. This pressure combines with the demands of work, leading me to having feelings of exhaustion and self-doubt about my ability to be a good mother. (Participant F8)

## Discussion

This study explored the perceptions of Latin American parents regarding the joys and challenges of parenthood; how their role was shaped by Marianismo and Machismo expectations, and how these experiences influence their overall wellbeing. Three core themes emerged from the thematic analysis conducted and will be discussed in light with scientific research around parenthood with European and US samples, given the scarcity of studies with Latin American parents. However, at the same time, this highlights the unique contribution of the present study. The first theme, “*Socioeconomic Pressures and Constraints on Parenting*” highlights the significant influence of socioeconomic factors on parenting experiences. Findings suggest that fathers felt the influence and pressure of the “Macho” culture embedded in what a “good parent” must look like, in addition to reporting feeling pressured by family members to be a good parent which has been observed in other cultures (Haller & Hadler, 2006). The social pressure based on Machismo gender-role expectations, often manifested in expectations to work tirelessly, make more money and secure high-quality education, typically through private schooling for their children. This adds to European research around parental practices from a general perspective – although not focused on wellbeing – where Kosmicki (2017) found that cultural and social expectations surrounding parenting are embraced by parents. However, the limited sample size in the present study calls for further research with a larger and more representative population of Latin American parents to determine the generalizability of this finding. Further analysis within the first theme revealed that parents’ low salaries presented a significant challenge in providing their children with high-quality education, perceived as crucial for future children’s success and parents’ sense of self-satisfaction. This supports previous research in other cultural contexts around parental expectations (Crede et al., 2015; Urbina-Garcia et al., 2022) suggesting that parents tend to focus more on children’s academic achievement – potentially at the expense of a more holistic developmental approach encompassing emotional wellbeing.

The theme also highlights the impact of broader social issues on parenting experiences. Previous socio-educational research with Latin American samples but not focused on wellbeing (Alfaro-Beracoechea et al., 2018; Kosmicki, 2017), reveals that participants identify violence and high crime rates as significant challenges when securing their children’s future. These findings, however, appear to contradict other studies suggesting a degree of habituation to violence among some Latin American populations (Rojas & García-Vega, 2017; Terrazas-Carrillo et al., 2016). Nevertheless, these studies did not focus on parents’ wellbeing which is what our study is contributing to. Our findings suggest that whilst parents may be habituated to crime and violence, these social problems are still having an important impact on parents’ by experiencing negative emotions such as frustration and fear. Future research should aim to expand the scope of research to consider a wider range of Latin American countries, and explore what specific emotions are triggered by what specific social events/challenges.

Through the lens of Diener’s model of subjective wellbeing (Diener & Seligman, 2004), the first theme suggests that the social expectations perceived by parents are somehow shaped by Marianismo and Machismo. These seem to influence the definition of a “good parent” which appears to shape parents’ self-evaluation and thus satisfaction with their role. Prior research indicates that parents engage in self-evaluation (Lee, 2014), drawing on their own childhood experiences, preconceptions of ideal parenting, and comparisons to other parents. However, a surprising key factor in our study is that parents’ self-evaluation is based on the perception of the *need* to be the “man of the house” (notion fed by family expectations) and thus, secure high-quality formal schooling for their children. Mothers, on the other hand, seem to be expected to be in charge of the everyday duties of the house. This finding adds to current international research with non-Latin American samples that shows parents’ motivation to provide a *good* education (Coleman, 2018) and the sense of fulfilment associated with fulfilling this perceived parental duty (Nomaguchi & Milkie, 2020). From a eudaimonic perspective (Disabato et al., 2016), this theme suggests that parents’ self-realization seems to be influenced by their perceived success in providing for their children’s education -adding evidence to research in other countries (Bertone & Franchi, 2016; Huta & Waterman, 2014).

The second overarching theme, “*The Joys of Sharing and Witnessing Growth*” revealed the antithesis to the challenges identified in the first theme. Despite the aforementioned socioeconomic pressures, participants reported finding enjoyment in their role as parents. A key source of this pleasure stemmed from sharing and transmitting their own values to their children. Interestingly, results show that passing onto values rooted in Machismo were a source of pride for fathers such as ensuring children become “strong men” and that they can “look after” their mum (being a female). Whilst this adds to the international scientific literature conducted in other continents reporting the satisfaction parents derive from passing on values, practices, and morals to their offspring (Bengtson, 2017), previous studies have not focused on parents’ wellbeing. Our finding, however, appears to contradict quantitative studies in Africa, which suggest a stronger parental emphasis on providing formal schooling opportunities (Kapinga, 2014). Some of parents’ values and practices in our study – which also seem to make parents feel self-realized, seem to be significantly shaped by Marianismo and Machismo aspects of the Latin American culture. However, future research should examine additional aspects of parenthood Latin American parents find most pleasurable. The second theme also identified specific activities that parents reported finding enjoyable. Adding evidence to quantitative research from the US and Europe (Hajal & Paley, 2020), participants in our study expressed pleasure in showing affection to their children. However, such affective expression seemed to be mediated by cultural expectations and thus limited for men, but not for women. Results show that some fathers prevented themselves from showing affection as this is inconsistent with their role as the “man of the house”, who is expected to display strength and masculinity. For mothers, however, it is appropriate to show affection to children – in line with the expectations of Marianismo. International research suggests that some parents may struggle with displays of affection (Brumariu, 2015, particularly those with mental health concerns such as depression or social phobia (Malik et al., 2015). However, our study makes a unique contribution to the evidence from those from US and Europe, by showcasing the ability that parents have developed—despite the severe socio-economic inequalities and high rates of crime and violence, to experience positive emotions.

Parents reported satisfaction in witnessing their children’s growth and development, often linked to “*learnings*” they had acquired from school or expected behaviours as a boy or a girl. Participants particularly reported to be proud of boys engaging in rough and tumble play and showing “toughness”, whereas for girls, being “obedient” and “helping” others was a source of pride for parents. It is to note that research shows that some educational curricula in some Latin American countries (e.g., Mexico, Chile), still contain a heavy component of traditional gender roles rooted in the Machismo and Marianismo culture (Bastos et al., 2020), -despite current trends of gender equality in the 21^st^ century. This may help explain parents’ satisfaction given the alignment with family-based expectations around gender roles. This finding adds to the international literature with non-Latin American samples, additional evidence that parents value the “*learnings*” associated with schooling (Froiland, 2015; Mínguez, 2020). Nevertheless, being proud of gender-based roles emphasizing masculinity and femininity-related behaviours has been observed in sociological, educational and anthropological studies. However, this particular finding focused on how “*seeing*” the reproduction of masculine/feminine traits in children as a source of pride and joy, seems to be initial evidence in studies around wellbeing with LA samples. The limited sample size in the present study limits the generalizability of these findings. Future research employing an interpretivist approach could explore a broader range of activities that parents find pleasurable within the context of additional Latin American cultures.

The second theme offers insights into the emotional experiences of parents within the context of their relationships with their children. Through a combined lens of hedonic and eudaimonic wellbeing models, we can speculate that parents experience positive emotions when transmitting values and knowledge to their children. This aligns with previous research demonstrating parental happiness associated with their children’s personal flourishing (Sypnowich, 2020). The enjoyment reported by participants in showing affection to their children, likely stems from these positive emotions. However, our study did not explore specific emotions associated with this activity, which future research should focus on given that some parents seem to prevent themselves from showing affection since this does not match the cultural expectation as a “man” in a Macho culture. Emotional Intelligence research (Gottman et al., 2013) suggests that parents with greater emotional awareness are more likely to display affection towards their children. Conversely, parents with lower emotional intelligence may struggle to share positive emotions with their offspring (Cline & Fay, 2020), however cross-cultural studies show that emotions are culturally dependant. Hence, the emotions experienced by these participants may be rooted in LA cultural values and social norms. Watson and Tellegen’s (1985) theory posits the possibility of experiencing both positive and negative emotions simultaneously. Participants in this study seem to exemplify this principle by reporting feelings of both sadness and happiness—interestingly despite the prevailing macho culture in LatAm, whereby fathers are not expected to show emotions. Moreover, the satisfaction parents derive from witnessing their children’s growth and showing machismo/marianismo traits, aligns with a hedonistic perspective. From a eudaimonic perspective (Lu et al., 2015), parental satisfaction might be rooted in their personal definition of fulfilment. Hence, this finding highlights that parents’ personal definition of satisfaction seems to mediate the power of cultural expectations framed by Machismo-led and Marianismo-led expectations. Future research should focus on the parameters parents use to identify additional joyful experiences.

The third theme, “*The Burden of Idealised Parenting and its Impact on Wellbeing*” highlights the significant impact of societal and familial pressures on parents’ emotional wellbeing. Participants reported experiencing happiness when they felt they were fulfilling their parental roles effectively. Conversely, sadness arose when they perceived a lack of fulfilment based on their own expectations which seems to be influenced by the cultural expectations. This finding contributes to previous evidence from research with European samples suggesting that parental happiness can be contingent on social activities with their children and extended family (Golombok, 2015). A core aspect of this theme seems to be the constant *socio-cultural pressure* to be a “*good parent*” and the “*man of the house*” (for fathers) reflects the masculinity expected in the culture, and requires fathers, ensure their children’s academic success, particularly in formal schooling. These findings add additional evidence to the body of literature from other non-Latin countries (Dizon-Ross, 2019), revealing a general parental focus on academic achievement. However, previous studies have not focused on the strong role of Machismo in fathers linked to their wellbeing. We argue that this can be seen as another relevant contribution of our study. Within the Latin American context specifically, social expectations seem to dictate that children should receive a high-quality education—typically in private schools due to perceived deficiencies in public education systems (Cruces et al., 2014). From an eudaimonic perspective (Mann et al., 2013), these findings can be understood through the lens of goal-directed behaviour. Parents striving to secure what they perceive as a prosperous future for their children, experience a heightened sense of wellbeing when this goal is achieved. However, the pressure to provide such a high-quality education can lead to feelings of judgement and inadequacy when expectations are not met – especially in light of a Macho culture where the father is expected to “provide” for his family. This pressure from family members appears to negatively impact parents’ wellbeing, leading them to feel emotionally drained and undervalued in a Macho society that values masculine strength and self-sufficiency. This finding makes a unique contribution to international studies revealing a positive association between family support and parental self-efficacy, as well as a sense of belonging—although such studies used Canadian, Lithuanian, Hong Kongese, US and Japanese samples (Albanese et al., 2019; Weiss et al., 2013) and did not focus on parental wellbeing.

Through the lens of Watson and Tellegen’s (1985) circumplex model, the third theme allows us to understand the co-occurrence of stress, anxiety, happiness, and sadness reported by participants. These findings add to the existent literature further demonstrating parents’ capacity to experience both positive and negative affect simultaneously (Aassve et al., 2015; Balbo & Arpino, 2016; Branje, 2018). Interestingly, this suggests that parenthood may be a complex experience encompassing both positive and challenging aspects—especially when navigating challenges such as high unemployment or low salaries experienced by Latin American adults. Limited time with their children due to work commitments emerged as another factor influencing parental wellbeing. This lack of face-to-face interaction may lead parents to question their fulfilment of their roles, potentially impacting their sense of self-realization and, ultimately, wellbeing. Research suggests that a low sense of self-realization can trigger negative emotions (Singh et al., 2020), which may explain the negative affect reported by parents when they are unable to spend time with their children. Mothers, for example, reported to feel happy and proud when seeing their daughters being obedient and helping others as well as helping with the household duties. Mothers reported to feel they were fulfilling their job as mums -clearly denoting the influence of their children showing Marianismo-related traits in mothers’ sense of fulfilment. However, we speculate that Marianismo-led expectations seems to shape mothers’ self-perception of fulfilment which if not met, their wellbeing may decrease. The theme further highlights parental self-questioning, a key component of the eudaimonic perspective on self-assessment and subjective wellbeing (Ryff, 2013). However, research also indicates that self-questioning can undermine parental self-efficacy (van Ingen et al., 2015), which has been associated with lower well-being (Giallo et al., 2013). Therefore, we speculate that with our sample, parental judgement or feelings of inadequacy may lead to a decline in wellbeing in parents.

The present study aimed to explore the perceptions of Latin American parents regarding what they enjoy the most and the main obstacles of parenthood shaped by the LA culture. We also aimed at exploring how these experiences influence their wellbeing. Our four key research questions were addressed as follows: a) results show that becoming a parent can trigger the experience of both, positive and negative emotions. These seem to be rooted in their culturally dependant perception of fulfilment and self-realization shaped by Machismo and Marianismo; b) results highlight that there are significant challenges faced by LA parents, including social and familial pressure to succeed, coupled with socio-economic inequalities and high rates of crime and violence prevalent in the region; c) findings suggest that transmitting values, showing affection to children, witnessing children’s growth and seeing expected machismo/marianismo traits in children, seem to be key sources of enjoyment for parents; d) findings suggest that socio-economic challenges may trigger negative emotions and prevent parents from fully enjoying parenthood; and Marianismo/Machismo-led expectations—observed in their children—seem to influence parents’ wellbeing in a positive way. These findings offer additional evidence to Asian and European studies with non-LA samples, and offer a unique perspective into the lived experiences of parents—heavily shaped by aspects of the Latin American culture. However, future research should consider individual differences, such as parental personality and intelligence, while also focusing on parental meaning-making processes. Exploring further the link between cultural expectations and parents’ wellbeing as well as and emotional experiences of parents is warranted. Including specific national variations within the broader category of “*Latin American*” parents, would allow for a more comprehensive understanding of this population.

### Strengths and limitations

The relatively small sample size restricts the generalizability of the findings. Future research should employ larger samples to identify additional trends and enhance the generalizability of results. The use of only eight open-ended questions limited the scope of the data collected. Future studies will benefit from including a wider range of questions encompassing additional factors, such as the exploration of parental emotions. The focus on parents from only seven Latin American countries limits our understanding of the diverse sociocultural factors at play across the region. Future research should incorporate a broader range of nationalities to provide a more comprehensive picture. The subjective nature of qualitative analysis inherently introduces the potential for personal biases to influence data interpretation. However, as discussed above and to address this, we made use of the bracketing technique in addition to critically discussing our analysis and findings with two Latin American colleagues specialized in parenthood and positive psychology. This helped use to corroborate our analysis and ensure cultural fidelity, reduce personal biases, and improve the credibility and trustworthiness of the findings. The cross-sectional design employed in this study limits our ability to understand the developmental nature of parental experiences and wellbeing. Longitudinal designs will be beneficial in future research to capture these changes over time. However, this study also offers unique insights. Our study stands as one of the first research to focus on LA parents’ perceptions regarding parenthood and wellbeing. By adopting an interpretivist perspective, the study offers initial evidence concerning parental wellbeing from the parents’ own viewpoints, contrasting with a more positivist approach. This research adds evidence to the growing body of scientific literature on parental wellbeing and the influence of parenthood.

### Implications for research and practice

There are few practical implications to highlight. Firstly, our findings could help inform educational policies to include activities to foster positive social connections and promote supportive relationships in schools. Secondly, by knowing what influences parents’ wellbeing, policymakers can develop psycho-educational interventions to offer parents effective strategies to navigate through parenthood and yet, be able to support their own wellbeing. Thirdly, recognizing sources of stress and sadness for parents could inform psychological interventions (perhaps provided by schools or local cities) to mitigate these negative experiences in parents—which could influence their parenting practices. Policies promoting economic equity in LA countries and access to quality education could contribute to parental wellbeing. Finally, our findings could be used to develop parental guidelines. These will help them acquire effective practices to promote not only their child’s wellbeing but also look after their own wellbeing. Policy modifications to help parents to look after their own wellbeing, could lead to a decrease in mental health problems in parents.

### Reflexivity

The researcher’s upbringing in Mexico and familiarity with Latin American cultural constructs like Machismo and Marianismo may have influenced his interpretation of the data, potentially leading to overemphasis on cultural aspects. However, his prolonged exposure to European culture and research approaches have shaped a critical lens towards traditional gender roles, lowering the impact of potential implicit bias during data collection and analysis. The researcher mitigated these risks by adopting an interpretative approach, maintaining transparency in collecting, coding and analysing the data. The bracketing technique was used as described above. He consulted two Latin American colleagues specialized in parenthood and positive psychology to ensure cultural fidelity and reduce personal biases. Recruitment was conducted with neutrality, avoiding favouritism towards participants who aligned with the researcher’s perspectives or experiences.

## Conclusions

The present study shows the multifactorial nature of parenthood in Latin America. Our findings depict parenthood as an experience that can be simultaneously stressful, demanding, and rewarding. Undoubtedly, the sociocultural context (heavily influenced by Marianismo and Machismo) plays a significant role in shaping parental experiences and, consequently, their wellbeing. Whilst parenthood seems to be a stressful role, parents can still find gratification in transmitting values to their children; and witnessing the acquisition of knowledge/skills and expected gender-based roles. Social aspects within Latin American countries, such as crime and violence, were identified as factors negatively influencing parental wellbeing. Social and familial pressures, alongside parental preconceptions of what constitutes “good parenting,” also emerged as significant influences.
